# Association between serum vitamin D and refractive status in United States adolescents: A cross-sectional study

**DOI:** 10.3389/fnut.2022.1038963

**Published:** 2022-12-08

**Authors:** Yanqing Li, Pengcheng Hu, Xianhui Wu, Qian Zou, Xiaozhu Liu, Jialing Liu, Yuxian Fu

**Affiliations:** ^1^Department of Ophthalmology, The Second Affiliated Hospital of Chongqing Medical University, Chongqing, China; ^2^Department of Ophthalmology, Chongqing Health Center for Women and Children, Chongqing, China; ^3^Department of Cardiology, The Second Affiliated Hospital of Chongqing Medical University, Chongqing, China; ^4^Department of Phase I Clinical Trial Center, The Second Affiliated Hospital of Chongqing Medical University, Chongqing, China

**Keywords:** vitamin D, refractive status, spherical equivalent, adolescent, NHANES

## Abstract

**Purpose:**

We performed this study to determine the relationship between serum vitamin D levels and refractive status in adolescents aged 12–19 years.

**Methods:**

Cross-sectional study using the National Health and Nutrition Examination Survey (NHANES) database from 2001 to 2006. We used weighted multivariate linear regression models to assess the association between serum vitamin levels and adolescent refractive status and then built a smooth curve fitting to investigate their internal non-linear relationships. Finally, subgroup analysis was performed according to gender, and the threshold effect of serum vitamin D levels on spherical equivalent degree was analyzed using a two-piecewise linear regression model.

**Result:**

A total of 5,901 adolescents aged 12 to 19 years were included in this study. After adjusting for all confounding factors, the multiple linear regression model showed no significant correlation between adolescent spherical equivalent degree and serum vitamin D [0.0019 (−0.0018, 0.0046)]. However, smooth curve fitting analysis showed an inverted U-shaped curve relationship between spherical equivalent degree and serum vitamin D levels in adolescents (turning point: 58.1 nmol/L). In analyses by gender subgroup, this inverted U-shaped relationship was found to be more pronounced in female adolescents (turning point: 61.6 nmol/L).

**Conclusion:**

Our results suggest that the correlation between refractive status and serum vitamin D in adolescents differs by gender. When serum vitamin D concentrations were <61.6 nmol/L in female adolescents and <53.2 nmol/L in male adolescents, the spherical equivalent degree showed a positive correlation with serum vitamin D levels. However, there was no significant correlation when adolescent vitamin levels exceeded this threshold.

## Introduction

When light passes *via* an interface between two media with different refractive indices, the light is deflected. This optical phenomenon is known as refraction. The refractive status of the eye is further separated into emmetropia and refractive error (myopia, hyperopia) based on the different refractive powers. Refractive error is the major cause of vision impairment and the second largest cause of blindness around the world (1). Currently, myopia affects 28.3% of the world’s population, with high myopia affecting 4.0% of the world’s population. By 2050, it is estimated that the prevalence of myopia will rise to 49.8% and the prevalence of high myopia will rise to 9.2% (2). Patients with high myopia are more likely to develop myopia-related eye diseases (myopic macular degeneration, retinal detachment, etc.) (3). Consequently, safeguarding eye health is a public health concern that all people must address. It is vital to establish rational and scientific medical interventions to prevent myopia’s progression.

Childhood and adolescence are crucial periods for developing myopia, as well as for its prevention and treatment. Environmental factors influence the development of myopia in addition to hereditary factors. Several medical studies have demonstrated the efficacy of increasing outdoor activities in preventing myopia in children (4, 5). However, the beneficial effect may not be contingent on the outside activity itself, but rather on the increase in sun exposure time (6). Clinically, it is considered that the negative link between sunshine exposure and myopia prevalence is associated with the vitamin D route in serum (7). Serum vitamin D is regarded as an objective biomarker for measuring outdoor time. Children who spent more time outdoors had greater levels of total vitamin D and D3 (8). Under exposure to sunlight, UVB stimulates the synthesis of vitamin D2 (Ergocalciferol) and vitamin D3 (Cholecalciferol) from ergosterol and 7-dehydrocholesterol (9), which together comprise blood vitamin D. Enzymatic hydroxylation converts vitamin D2 and D3 to 25(OH)D and then to 1,25-dihydroxy vitamin D2 or D3 (calcitriol) (10). The Dietary Guidelines for Vitamin D assess serum vitamin D levels using serum 25(OH)D concentrations (11). Serum 25(OH)D concentrations <50 nmol/L are defined as vitamin D deficiency (12).

A study based on the Korean National Health and Nutrition Examination Survey (KNHANES) database revealed a negative association between serum 25(OH)D levels and myopia in Korean adolescents (13). Gareth et al. (14) examined the connection between 25(OH)D concentrations at several ages and the risk of myopia at age 20 by measuring serum 25(OH)D concentrations in adolescents at the follow-up ages of 6, 14, 17, and 20. According to research, serum 25(OH)D concentrations decrease dramatically before the onset of myopia. The lower the serum 25(OH)D levels in adolescents at 17 and 20 years of age, the higher the likelihood of myopia at 20 years of age. The meta-analysis of seven studies conducted by Tang et al. (15) revealed that mean 25(OH)D concentrations were considerably lower in the myopic group than in the non-myopic group; higher 25(OH)D concentrations significantly reduced the likelihood of myopia. In a subgroup analysis by age, however, it was determined that there was a significant association between vitamin D levels and myopia in people over 18 years of age, whereas there was no significant association in participants under 18 years of age. In both investigations, the sample size of adolescents (12 to 18 years old) was insufficient to analyze the association between refractive status and vitamin D levels in this age range with precision.

Currently, the influence of serum vitamin D on adolescents’ refractive status remains debatable. Several studies (16, 17) suggest that lower serum 25(OH)D3 concentrations are related to an increased risk of myopia; however, the average age of participants in these studies was over 20 years. Myopia is prevalent during childhood and adolescence, with the average age of stabilization being between 15 and 16 years (18). It is unknown whether elevated serum vitamin D levels during this period promote changes in the spherical equivalent. To investigate the relationship between vitamin D and adolescent visual development, we use the NHANES database to explore the possible association between vitamin D levels and refractive status in adolescents in the United States using a cross-sectional study with a large sample size to provide new clinical ideas for myopia prevention.

## Materials and methods

### Study population

In this study, data from the National Health and Nutrition Examination Survey (NHANES), a population-based cross-sectional survey, and a national study conducted by the National Center for Health Statistics (NCHS) of a nationally representative stratified sample based on interviews and medical examinations, with data released biennially, were analyzed. We pooled data from three 2-year NHANES cycles completed between 2001 and 2006. All participants provided their written informed consent (age ≥ 18 years on their own; age < 18 years on their parents/guardians), and the NCHS Ethics Review Committee approved the conduct of NHANES.

There were 31,509 participants in the NHANES database from 2001 to 2006. After excluding data on missing refractive status (*N* = 12,047), missing vitamin D levels (*N* = 1,239), age > 19 years (*N* = 12,255), history of previous refractive surgery (*N* = 6), history of cataract surgery (*N* = 1), unclear history of previous eye surgery (*N* = 11), and history of clearly diagnosed diabetes (*N* = 29) or unknown history of diabetes (*N* = 20), 5,901 patients were finally included for regression analysis.

### Study variables

In this study, Serum vitamin D levels were the primary independent variable, and the dependent variable was the change in adolescent spherical equivalent degree. All participants’ serum 25(OH)D levels were evaluated using a radioimmunoassay kit (DiaSorin, Stillwater, Minnesota, USA). The Nidek ARK-760 autorefractor acquired objective refraction data in a non-dilated state. In the analysis, the spherical equivalent degree (SER, the average refraction of the two major meridians) was calculated using the right eyes of all participants. Only participants with visual acuity of 20/30 or worse had their distance visual acuity reevaluated using objective-automated optometry. Participants with full blindness or inability to see in both eyes were also excluded, as were those with severe infection (purulent discharge, redness, and inflammation) in one or both eyes.

Additionally, the following covariates were included: age, sex, race, household income, education, body mass index, blood calcium, serum phosphorus, and the time of vitamin D assay. Age was obtained by asking the participant’s age or date of birth in an interview. This race/ethnicity variable is derived by combining responses to questions on race and Hispanic origin, there are five races as follows: Mexican American, Other Hispanic, Non-Hispanic White, Non-Hispanic Black, and Other Race. After the information about sources of income was obtained in the Family Interview Income section questionnaire, the respondents were asked to report the total combined family income for themselves and the other members of their family in dollars. Educational attainment was obtained by interviewing information on the highest level of education completed by participants aged 6–19 years. BMI is defined as the weight (kg)/height^2^ (m). The LX20 system uses indirect (or diluted) ISE methodology to measure calcium concentration in serum, plasma, or urine. The LX system uses a timed-rate method to determine the concentration of phosphorus in serum, plasma, and urine. Serum vitamin D levels were measured over two time periods: November 1 through April 30 and May 1 through October 31.

### Statistical analyses

To compare the demographic characteristics of the number of people included in this study, categorical variables were expressed as frequencies or percentages, and continuous variables were expressed as mean ± standard deviation. Weighted linear regression models (continuous variables) and weighted chi-square tests (categorical variables) were also used to calculate group differences. *p* < 0.05 was statistically significant.

A weighted multiple regression model was applied to analyze the independent correlation between vitamin D and spherical equivalent degree, and Weighted generalized additive models and smooth curve fittings were employed to address the non-linearity of serum vitamin D levels and spherical equivalent degree. Subgroup analysis was performed according to gender, and the threshold effect of serum vitamin D levels in spherical equivalent degree was analyzed using a two-piecewise linear regression model.

All analyses were performed using the R language.

## Results

The sociodemographic and medical features of the subjects are detailed in Table 1. This study comprised 5,901 individuals. Table 1 displays the results of the quadruple classification grouping (Q1–Q4) according to different serum 25(OH)D levels. There were substantial variations between the groups in terms of age, sex, race, education, body mass index, blood calcium, and serum phosphorus. Serum 25(OH)D levels were greater in male adolescents (64.07 **±** 20.68 nmol/L) than in female adolescents (62.16 **±** 24.12 nmol/L) when categorized according to gender (see Supplementary material for details).

**TABLE 1:** Description of 5,901 participants.

Vitamin D	Q1	Q2	Q3	Q4	*P*-value
Age (years)					<0.0001
12–15	41.47	54.17	55.89	49.51	
16–19	58.53	45.83	44.11	50.49	
Gender (%)					<0.0001
Male	39.51	49.91	53.30	53.70	
Female	60.49	50.09	46.70	46.30	
Race (%)					<0.0001
Mexican American	15.37	18.04	13.61	5.55	
Other Hispanic	3.22	8.89	8.22	3.21	
Non-Hispanic White	15.24	42.01	66.16	85.28	
Non-Hispanic Black	54.39	23.77	7.80	2.56	
Other Race	11.79	7.30	4.22	3.40	
Income poverty ratio	1.89 ± 1.46	2.25 ± 1.59	2.66 ± 1.63	2.94 ± 1.57	<0.0001
Education					<0.0001
Less Than 9th Grade	37.49	49.10	48.45	40.97	
9th Grade or higher	62.51	50.90	51.55	59.03	
Exam month (%)					<0.0001
May 1 through October 31	35.39	44.46	56.60	71.65	
November 1 through April 30	64.61	55.54	43.40	28.35	
BMI (kg/m^2^)	26.01 ± 7.43	24.34 ± 5.74	23.39 ± 5.51	22.30 ± 4.59	<0.0001
Calcium (mmol/L)	2.40 ± 0.08	2.43 ± 0.08	2.44 ± 0.07	2.44 ± 0.08	<0.0001
Phosphorus (mmol/L)	1.38 ± 0.21	1.44 ± 0.22	1.44 ± 0.21	1.43 ± 0.21	<0.0001
Sphere	−1.31 ± 2.01	−1.28 ± 2.14	−1.09 ± 1.84	−1.05 ± 1.84	0.0007
Cylinder	0.69 ± 0.73	0.63 ± 0.66	0.60 ± 0.61	0.56 ± 0.55	<0.0001
Spherical equivalent (D)	−0.96 ± 1.91	−0.96 ± 2.03	−0.79 ± 1.77	−0.77 ± 1.78	0.0073

Continuous variables were represented by Mean + SD, *P*-value was calculated by weighted linear regression model.

Categorical variables calculated by %, *P*-value was calculated by weighted chi-square test.

The quadruple classification grouping according to different serum 25(OH)D levels: Q1(9.1–38.5 nmol/L); Q2(39.7–51.6 nmol/L); Q3 (51.9–65.8 nmol/L); Q4 (66.5–180 nmol/L).

Table 2 contains three weighted univariate and multivariate linear regression models: Model 1 is unadjusted; model 2 is adjusted for age, sex, and race/ethnicity; and model 3 is adjusted for all covariates (age, sex, race/ethnicity, household income, educational attainment, body mass index, blood calcium, serum phosphorus, exam month, etc.). In the unadjusted model, there was no connection between the adolescent spherical equivalent degree and serum 25(OH)D [0.0020 (−0.0001, 0.0041)]. Model 3 similarly indicated no significant link between teenage spherical equivalent degree and serum 25(OH)D after controlling for all covariates [0.0019 (−0.0018, 0.0046)]. Stratified by race and vitamin D levels, it was discovered that only when vitamin D levels were in Q3 (51.9–65.8 nmol/L) [0.2245 (0.0338, 0.4152)] and Q4 (66.5–180 nmol/L) [0.2488 (0.0527, 0.4449)], did adolescent spherical equivalent degree demonstrate a positive association with blood 25(OH)D levels.

**TABLE 2:** The association between Vitamin D (nmol/L) and spherical equivalent (D).

	Non-adjusted, β (95% CI)	Adjust I, β (95% CI)	Adjust II, β (95% CI)
Vitamin D (nmol/L)	0.0020 (−0.0001, 0.0041)	0.0018 (−0.0006, 0.0042)	0.0019 (−0.0018, 0.0046)
**Stratified by gender**			
Male	0.0037 (0.0006, 0.0069)	0.0043 (0.0006, 0.0079)	0.0026 (−0.0014, 0.0065)
Female	0.0006 (−0.0022, 0.0034)	−0.0005 (−0.0038, 0.0028)	0.0004 (−0.0031, 0.0040)
**Stratified by race**			
Mexican American	0.0048 (−0.0003, 0.0098)	0.0043 (−0.0008, 0.0095)	0.0043 (−0.0014, 0.0101)
Other Hispanic	0.0096 (−0.0031, 0.0223)	0.0069 (−0.0059, 0.0197)	0.0070 (−0.0070, 0.0209)
Non-Hispanic White	−0.0003 (−0.0046, 0.0039)	−0.0001 (−0.0043, 0.0041)	−0.0000 (−0.0047, 0.0047)
Non-Hispanic Black	0.0051 (−0.0004, 0.0107)	0.0043 (−0.0015, 0.0100)	0.0039 (−0.0023, 0.0100)
Other race	0.0152 (0.0014, 0.0290)	0.0148 (0.0007, 0.0288)	0.0084 (−0.0068, 0.0235)
**Quintiles of vitamin D**			
Lowest quintile Q1 (9.1–38.5 nmol/L)	Reference	Reference	Reference
Q2 (39.7–51.6 nmol/L)	−0.0106 (−0.1827, 0.1614)	−0.0010 (−0.1816, 0.1797)	−0.0022 (−0.1922, 0.1877)
Q3 (51.9–65.8 nmol/L)	0.1505 (−0.0067, 0.3078)	0.1654 (−0.0133, 0.3440)	0.2245 (0.0338, 0.4152)
Q4 (66.5–180 nmol/L)	0.1799 (0.0297, 0.3301)	0.1997 (0.0200, 0.3794)	0.2488 (0.0527, 0.4449)
*P* for trend	0.002	0.005	0.001

Non-adjusted model adjust for: none.

Adjust I model adjust for: gender, age, race.

Adjust II model adjust for: gender, age, race, education level, income poverty ratio, calcium, phosphorus, BMI, exam month.

**FIGURE 1:**
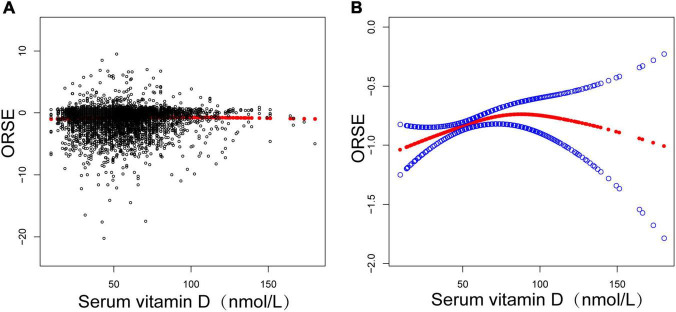
**(A)** Scatterplot: Each black point represents a sample. **(B)** Red solid arcs indicate the smoothed curve fit between variables. The area between two blue dotted lines is expressed as a 95% CI. Adjusted for age, sex, race, household income, education, body mass index, blood calcium, serum phosphorus, and other covariates.

**FIGURE 2:**
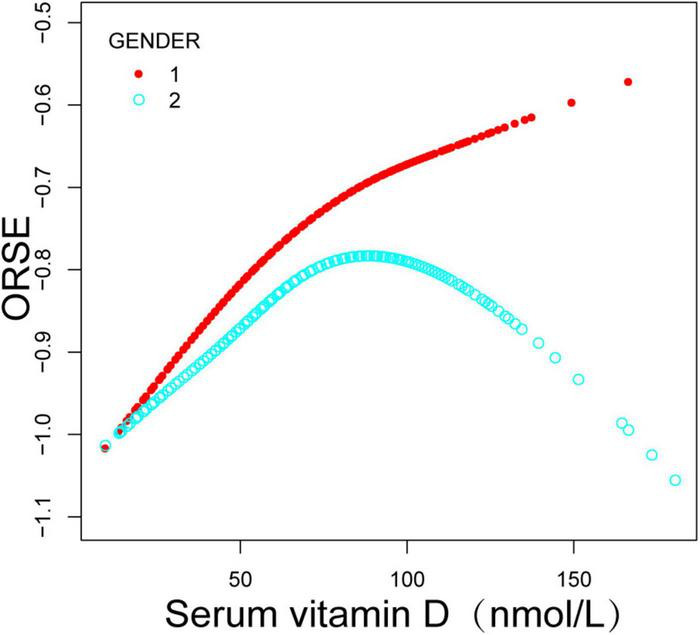
Relationship between refractive status and serum vitamin D in adolescents, stratified by sex. Adjustments were made for covariates such as age, race, household income, education, body mass index, serum calcium, serum phosphorus and other covariates.

Additionally, after controlling for all factors, this study discovered a non-linear association between adolescent refractive status and serum vitamin D levels by smooth curve fittings, an inverted U-shaped curve (Figure 1 and Table 3 turning point: 58.1 nmol/L). Lastly, subgroup analysis by gender revealed that this inverted U-shaped relationship was only present in female adolescents (Figure 2 and Table 4, turning point: 61.6 nmol/L). There was a positive correlation between refractive status and vitamin D levels when serum vitamin D levels were <61.6 nmol/L [0.0087 (0.0007, 0.0166)], but there was no significant correlation when serum vitamin D levels were >61.6 nmol/L [−0.0036 (−0.0085, 0.0014)]. Certain saturation versus threshold effects was also observed in male adolescents (Figure 2 and Table 5, turning point: 53.2 nmol/L), with a positive correlation between their refractive status and vitamin D levels when serum vitamin D levels were <53.2 nmol/L [0.0145 (0.0028, 0.0261)], but with serum vitamin D levels >53.2 nmol/L, the refractive status did not correlate significantly with vitamin D levels [−0.0003 (−0.0051, 0.0044)].

**TABLE 3:** Threshold effect analysis of vitamin d on spherical equivalent using the two piecewise linear regression model.

Spherical equivalent (D)	Adjusted β (95% CI)
Fitting by the standard linear model	0.0019 (−0.0018, 0.0046)
**Fitting by the two-piecewise linear model**	
Inflection point	58.1
Vitamin D (nmol/L) < 58.1 (nmol/L)	0.0106 (0.0040, 0.0172)
Vitamin D (nmol/L) > 58.1 (nmol/L)	−0.0013 (−0.0048, 0.0021)
Log likelihood ratio	0.005

**TABLE 4:** Threshold effect analysis of vitamin d on spherical equivalent in female using the two piecewise linear regression model.

Spherical equivalent (D)	Adjusted β (95% CI)
Fitting by the standard linear model	0.0004 (−0.0031, 0.0040)
**Fitting by the two-piecewise linear model**	
Inflection point	61.6
Vitamin D (nmol/L) < 61.6 (nmol/L)	0.0087 (0.0007, 0.0166)
Vitamin D (nmol/L) > 61.6 (nmol/L)	−0.0036 (−0.0085, 0.0014)
Log likelihood ratio	0.023

**TABLE 5:** Threshold effect analysis of vitamin D on spherical equivalent in male using the two piecewise linear regression model.

Spherical equivalent (D)	Adjusted β (95% CI)
Fitting by the standard linear model	0.0026 (−0.0014, 0.0065)
**Fitting by the two-piecewise linear model**	
Inflection point	53.2
Vitamin D (nmol/L) < 53.2 (nmol/L)	0.0145 (0.0028, 0.0261)
Vitamin D (nmol/L) > 53.2 (nmol/L)	−0.0003 (−0.0051, 0.0044)
Log likelihood ratio	0.033

## Discussion

The major purpose of this large cross-sectional investigation was to determine whether the adolescent refractive status is independently linked with serum vitamin D levels using data from a nationally representative U.S. sample. The association between refractive status and blood vitamin D levels in teenagers was shown to be non-linear, with an inverted U-shaped curve (turning point: 58.1 nmol/L). In addition, gender-related confounding factors were taken into consideration. Subgroup analysis for gender revealed that this inverted U-shaped relationship was more pronounced among female adolescents and that there were significant turning points. When serum vitamin D levels were less than 61.6 nmol/L, the spherical equivalent degree was favorably connected with vitamin D levels, but the connection was not significant when serum vitamin D levels were >61.6 nmol/L. Male adolescents similarly revealed a saturation threshold effect, with a positive connection between spherical equivalent and vitamin D levels when serum vitamin D levels were less than 53.2 nmol/L. When their serum vitamin D levels exceeded 53.2 nmol/L, this favorable connection was likewise insignificant.

Previous articles have examined the relationship between serum 25(OH)D levels and myopia. Studies have indicated a negative correlation between serum 25(OH)D levels and myopia (19). In a prospective population-based cohort study (20) involving 2,666 6-year-old children, after adjusting for confounding factors such as time spent outdoors, it was discovered that children with lower serum 25(OH)D levels had longer eye axis length, whereas those with higher 25(OH)D had a lower risk of myopia. However, Guggenheim et al. were denied and found that serum vitamin levels were not substantially associated with the onset of myopia (8). Chan et al. (21) conducted a systematic review, and the final results of their meta-analysis supported this position. A recent Mendelian randomization study demonstrated no association between DHCR7, CYP2R1, GC, and CYP24A1 gene-determined 25(OH)D levels and myopia severity (22). There are inconsistencies in these clinical research findings. This is due to flaws in the trial design of some of the research, and the linear regression analysis may obscure the true link between the two. Moreover, changes in demographic factors, sample size, serum 25 (OH) D levels of participants, and statistical methodologies for outdoor exercise time in the study may influence the outcomes.

Limited clinical data exist to date addressing the independent influence of serum vitamin D on the spherical equivalent degree. In a survey of 186 Chinese children, Fan et al. (23) discovered a favorable connection between serum 25(OH)D levels and a spherical equivalent degree in teenagers. In children with serum 25(OH)D deficit, the prevalence of moderate and extreme myopia was 2.051 times higher than in those with serum 25(OH)D sufficiency. For every 1 ng/ml increase in serum vitamin D content, the spherical equivalent degree dropped by 0.01 D in Korean adults, according to Jung and Jee (17). Nevertheless, in a separate study (24), also based on the NHANCE database, 6,855 participants aged 12 to 25 were recruited between 2003 and 2008 Based on the spherical equivalent of −0.75D, the patients were separated into two groups: non-myopic and myopic. Serum vitamin D levels were comparable between the two groups, and there was no significant relationship between serum 25[OH]D levels and spherical equivalent degree. In contrast, we chose the same study population but used different study designs and data analysis techniques. We also discovered no significant link between refractive status and serum vitamin D levels in teenagers in the weighted multivariate linear regression models. Using smooth curve fittings, we discovered that the relationship was not a simple linear, but rather an inverted “U” curve with a significant threshold. There was a positive correlation between the spherical equivalent lens and vitamin D level when the serum vitamin D level was <58.1 nmol/L. This shows that a normal vitamin D concentration may have a moderating influence on adolescents’ refractive status.

There are numerous hypotheses regarding the mechanism of vitamin D’s effect on its refractive condition. Vitamin D has been shown to regulate the expression of dopaminergic-related genes (25) or enhance VDR (vitamin D receptor)-RAR (retinoic acid receptor) heterodimer-mediated gene expression (26), thereby mediating retinal dopamine (27) and retinoic acid (RA) (28) to control the development of myopia by regulating the growth of the eye axis. In addition, the cornea may contain vitamin D receptors (VDRS) (29) and vitamin D hydroxylase. Vitamin D supplementation considerably increases the levels of 25(OH)D3 and 24,25(OH)_2_D3 in tear and atrial fluid (30), with 25(OH)D3 enhancing the barrier function of ocular epithelial cells (31). McMillan et al. (32) discovered that vitamin D deficiency can result in irregular astigmatism in the central corneal optical zone and that vitamin D supplementation can improve the mechanical properties of the cornea, thereby reversing a certain degree of refractive state and “emmetropizing” myopia. Annamaneni et al. (33) observed that despite the proximity of the VDR gene to a myopia-associated locus (MYP-3), they did not identify a direct influence on the development of myopia. It may minimize the risk of myopia indirectly by increasing mineral stability and modulating the function of the ciliary muscle. Further research is necessary to determine whether the regulating effects of vitamin D on spherical equivalent are mediated by vitamin D receptors (VDRS).

There is evidence that gender is an independent factor linked with blood 25(OH)D concentrations, with significantly lower vitamin D levels in women than in men (34). Our data bolster this viewpoint. Sex hormones throughout teenage development may be a gender-related factor that influences the regulation and stabilization of adolescents’ serum vitamin D levels. Testosterone levels are favorably connected with vitamin D levels in men (35), whereas estrogen levels stimulate vitamin D accumulation and increase the expression of vitamin D receptors in women (36). Additionally, it has been discovered (34, 37) that subcutaneous adipose tissue retains significant doses of vitamin D and can locally metabolize 25(OH)D. Although this study did not uncover significant variations in BMI between male and female adolescents, it does not rule out the possibility that changes in subcutaneous fat thickness between males and females could influence serum vitamin D levels. Furthermore, we found that the correlation between refractive status and serum vitamin D in adolescents differed by gender, with different threshold effects. The study by Annamaneni et al. (33) indirectly explains our results at the genetic level. The f allele produced by the VDR gene Fok1 (exon 2 start codon) may enhance the sex-specific risk of myopia onset and progression in women, particularly those with modest myopia. However, additional cohort studies with large samples are required to investigate the role of gender on the association between the two.

Based on a wide sample of data, this study investigated the linear association between refractive status and serum vitamin D levels in teenagers. The NHANES database collects a representative sample of U.S. inhabitants using a stratified, multistage sampling strategy. Standardized procedures were followed for data collection. The sampling strategy involved four steps: counties, segments, households, and individuals. To reduce the likelihood of selection bias, all our studies are based on weighted data. Therefore, it may be said that the present study’s findings have some generalizability. Nonetheless, this study has numerous obvious drawbacks. First, because the NHANES 2001–2006 database did not contain information on participants’ duration of UVB exposure and vitamin D intake was assessed. Therefore, it is plausible that differences in outdoor exercise and diet may affect the serum vitamin levels of the individuals. Second, cycloplegic optometry was not performed on the patients included in this database. This may result in a higher result than the real refraction due to pseudo myopia refraction. However, it has also been indicated that non-ciliary muscle paralysis has a smaller impact on the confounding of the regression model (38). Thirdly, changes in teenagers’ refractive status are an ongoing process that is impacted by numerous factors, such as the presence of myopic parents, the amount of time spent exercising outside, and the amount of time spent reading daily, which may weaken the observed relationships. Fourth, because the study based on the NHANES database is a cross-sectional study, a causal relationship cannot be established between changes in refractive status and changes in serum vitamin D levels.

## Conclusion

In conclusion, our investigation revealed a non-linear association between refractive status and blood vitamin D levels in teenagers, represented by an inverted U-shaped curve. And the association between the two varied by gender. There was a positive link between adolescent spherical equivalent degree and normal concentrations of serum vitamin D (53.2 nmol/L in male adolescents and 61.6 nmol/L in female adolescents), which may have a moderating influence on the refractive status of adolescents. When vitamin D levels exceeded this threshold, there was no significant relationship with an adolescent spherical equivalent degree, suggesting that vitamin D may not have a protective impact in this circumstance. In this investigation, however, causality could not be established. Future gender-specific prospective cohort studies are required to clarify the causal link between blood vitamin D levels and refractive status in adolescents and to discover the physiological processes underlying this conclusion. This helps us to better understand if vitamin D-based prevention techniques are gender-specific and can provide new insights for the prevention and control of myopia in teenagers.

## Data availability statement

The datasets presented in this study can be found in online repositories. The names of the repository/repositories and accession number(s) can be found below: Our study relied on data from the NHANES database. The datasets used during the current study are available at https://www.cdc.gov/nchs/nhanes/ and https://pan.baidu.com/s/1vF566sXfzm0srz2z9_zwTQ?pwd=oz2u, code is oz2u.

## Ethics statement

All participants provided their written informed consent (age ≥ 18 years on their own; age < 18 years on their parents/guardians) and the National Centre for Health Statistics (NCHS) Ethics Review Committee approved the conduct of National Health and Nutrition Examination Survey (NHANES).

## Author contributions

YF: conceptualization. PH and JL: data curation and formal analysis. YL, XW, and QZ: writing – original draft preparation. YF, XL, YL, and PH: writing – review and editing. All authors have read and agreed to the published version of the manuscript.
