# Supplementary material for: Association between serum vitamin D and refractive status in United States adolescents: A cross-sectional study
Source: Front Nutr. 2022 Dec 8;9:1038963. doi: 10.3389/fnut.2022.1038963 (PMC9773557; doi:10.3389/fnut.2022.1038963)
Supplement: Supplementary file 1 [file Table_1.DOCX]

| ATTACHED LIST 1 \| Description of 5901 participants. | | | |
| --- | --- | --- | --- |
|  | Male(n=2974) | Female(n=2927) | P value |
| Income poverty ratio | 2.64 ± 1.62 | 2.55 ± 1.62 | 0.0451 |
| BMI (kg/m^2^) | 23.31 ± 5.47 | 23.59 ± 5.79 | 0.0620 |
| Calcium (mmol/L) | 2.45 ± 0.08 | 2.41 ± 0.08 | <0.0001 |
| Phosphorus (mmol/L) | 1.46 ± 0.23 | 1.39 ± 0.18 | <0.0001 |
| Sphere (D) | -1.13 ± 1.91 | -1.15 ± 1.93 | 0.7164 |
| Cylinder (D) | 0.63 ± 0.64 | 0.57 ± 0.59 | 0.0002 |
| Spherical equivalent (D) | -0.81 ± 1.81 | -0.86 ± 1.87 | 0.3201 |
| Vitamin D (nmol/L) | 64.07 ± 20.68 | 62.16 ± 24.12 | 0.0011 |
| Race (%) |  |  | 0.4814 |
| Mexican American | 11.55 | 11.15 |  |
| Other Hispanic | 5.38 | 5.94 |  |
| Non-Hispanic White | 62.86 | 63.26 |  |
| Non-Hispanic Black | 14.35 | 14.71 |  |
| Other Race | 5.86 | 4.94 |  |
| Exam month (%) |  |  | 0.1948 |
| May 1 through October 31 | 58.62 | 56.95 |  |
| November 1 through April 30 | 41.38 | 43.05 |  |
| Age (years) |  |  | 0.2779 |
| 12-15 | 50.44 | 51.86 |  |
| 16-19 | 49.56 | 48.14 |  |
| Education |  |  | 0.3018 |
| Less Than 9th Grade | 43.46 | 44.79 |  |
| 9th Grade or higher | 56.54 | 55.21 |  |
| Continuous variables were represented by Mean + SD，P value was calculated by weighted linear regression model.  Categorical variables calculated by %，P value was calculated by weighted chi-square test. | | | |
